# Extraction and surface modification of cellulose fibers and its reinforcement in starch-based film for packaging composites

**DOI:** 10.1186/s40643-023-00631-w

**Published:** 2023-01-25

**Authors:** Halimatun Saadiah Hafid, Farah Nadia Omar, Ezyana Kamal Bahrin, Minato Wakisaka

**Affiliations:** 1 Institute of Plantation Studies, Universiti Putra Malaysia, 43400 UPM Serdang, Selangor Malaysia; 2 Preparatory Center for Science and Technology (PPST), Universiti Malaysia Sabah, Jalan UMS, 88400 Kota Kinabalu, Sabah Malaysia; 3 Institute of Plantation Studies, Universiti Putra Malaysia, UPM Serdang, 43400 Selangor, Malaysia; 4 Centre of Foundation Studies for Agricultural Science, Universiti Putra Malaysia, UPM Serdang, 43400 Selangor, Malaysia; 5 Graduate School of Life Science and Systems Engineering, Kyushu Institute of Technology, 2-4 Hibikino, Wakamatsu-Ku, Kitakyushu, 808-0196 Japan

**Keywords:** Gloss art paper, Cellulose, Extraction, Ultrasonic, Surface modification, Mechanical properties

## Abstract

**Background:**

Cellulose extraction from gloss art paper (GAP) waste is a recycling strategy for the abundance of gloss art paper waste. Here, a study was conducted on the impact of ultrasonic homogenization for cellulose extraction from GAP waste to improve the particle size, crystallinity, and thermal stability.

**Results:**

At treatment temperature of 75.8 °C, ultrasonic power level of 70.3% and 1.4 h duration, cellulose with properties of 516.4 nm particle size, 71.5% crystallinity, and thermal stability of 355.2 °C were extracted. Surface modification of cellulose GAP waste with H_3_PO_4_ hydrolysis and 2,2,6,6-tetramethylpiperidine-1-oxyl radical (TEMPO) oxidation was done followed by starch reinforcement. Surface hydrophobicity and mechanical strength were increased for H_3_PO_4_ hydrolysis and TEMPO oxidation starch–cellulose. No reduction of thermal properties observed during the treatment, while increment of crystallinity index up to 47.65–59.6% was shown. Neat starch film was more transparent, followed by starch–TEMPO film and starch–H_3_PO_4_ film, due to better homogeneity.

**Conclusions:**

The cellulose GAP reinforced starch film shows potential in developing packaging materials and simultaneously provide an alternative solution of GAP waste recycling.

**Graphical Abstract:**

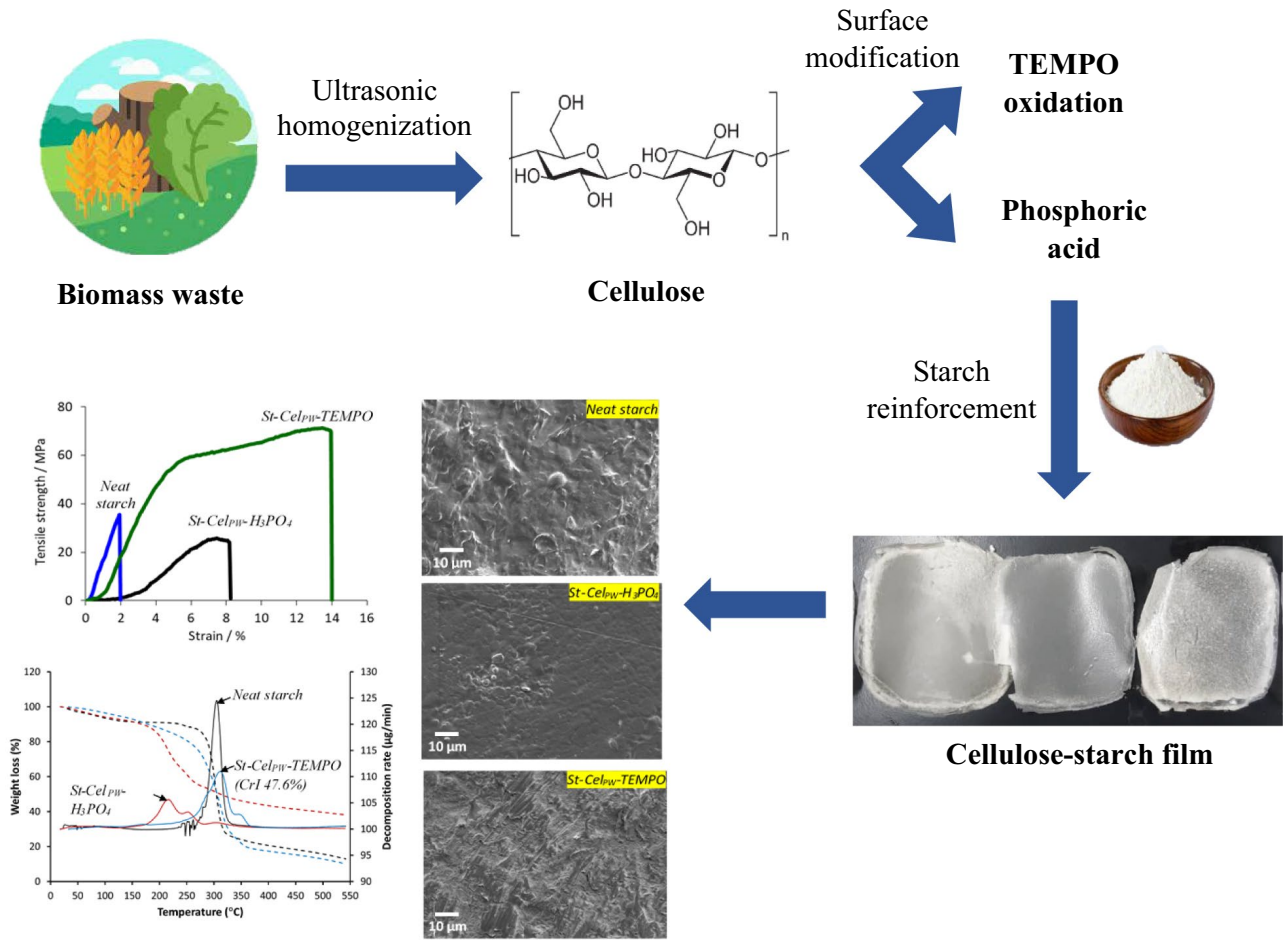

**Supplementary Information:**

The online version contains supplementary material available at 10.1186/s40643-023-00631-w.

## Introduction

Waste recycling is targeting to reduce the final disposal of waste in landfills and allow for partial recovery of raw materials for other manufacturing purposes (Basri et al. 2017; Tiew et al. 2019; Kuo et al. 2021). Paper waste is generated at 400 million tons per year in the form of paper and corrugated cardboard boxes in which the majority of the paper waste is being recycled or finally disposed in the landfills (Lin et al. 2020; Oliva et al. 2020). Recycling of paper is practiced in many countries; however, the lack of the clear regulation and quality control procedure led to improper recycling practices (Liu et al. 2020). Despite of its abundant in volume, the recycled paper is a composed of 70–80% of crystalline cellulose which is the stable polymers with good mechanical properties and has been shown as an ideal material for the sustainable production of high-value cellulose composite materials (Jiang et al. 2020; Liu et al. 2021).

Paper waste recycling involves mechanical and chemicals such as acid and alkali treatments at high temperature aiming to swell and loosen the cellulose fiber structure. However, this process may shorten and reduce the crystallinity of cellulose fiber resulting in low mechanical properties that led to inferior qualities of the composite products and did not meet the requirement of the customers (Lin et al. 2020; Ahmad Khorairi et al. 2021; Asem et al. 2021). Hence, the physical technique of ultrasonication homogenization process has been proposed to improve the cellulose extraction by increasing the cellulose dispersion and reduce the agglomeration of the cellulose for more stable suspension (Mohd Ishak et al. 2020). Meanwhile, the ultrasonication treatment is feasible in enhancing the accessibility and reactivity of cellulose (Qu et al. 2021). Ultrasonication homogenization treatment has been assisted with acid or alkaline in the extraction of cellulose from various lignocellulosic biomass such as wheat straw and oil palm bunch (Abdullah et al. 2016), maize stalk (Gu et al. 2020), and *Miscanthus x gigantis* grass (Singh et al. 2020). High-pressure ultrasonication homogenization provides energy of approximately 10–100 kJ/mol which can break down the hydrogen bond and disintegrate the cellulose polymers into microfibrillated cellulose bonding by altering its size and morphology (Mohd Ishak et al. 2020). The microbubbles generated by the ultrasonication process collapse during the compression cycle, producing high temperature and pressure which leads to the destruction of water molecules into free radicals of •O, •OH and HO_2_•. Highly reactive radicals created an impact in the liquid, applying forces from all sides to crush or fracture the cellulose, hemicellulose, and lignin networks (Singh et al. 2020). Ultrasonic homogenization treatment with the combination of heat and pressure is expected to expedite the dissolution rate of cellulose.

An improvement of surface properties increased cellulose functional performance in terms of mechanical strength, thermal properties, resistance against water, and improvement in wettability and hydrophobicity properties (Shahbazi et al. 2016; Mohamed et al. 2017). Cellulose with surface hydroxyl groups is hydrophilic and prone to water absorption which affects the barriers against gas and water vapors. Hence, the hydrophobicity properties of cellulose need to be improved through chemicals or composites (Balasubramaniam et al. 2020). Chemicals such as sulfuric acid and alkali were used for surface modification of cellulose (Zainal et al. 2019; Megashah et al. 2020). Later, the use of 2,2,6,6-tetramethylpiperidine-1-oxyl radical (TEMPO)-mediated oxidation showed promising results in modifying the cellulose surface as the TEMPO-oxidized cellulose fibers (TOCF) introduces negatively charged carboxyl groups on the surface of the cellulose and form homogeneous TOCF suspensions stabilized with surface charge via controlling the oxidant content (Qu et al. 2021; Zhou et al. 2021). The negatively charge of carboxylate group from the TEMPO oxidation on the cellulose surface would allow the dispersion of cellulose in water to form more stable suspension without agglomeration in certain period of time (Zhou et al. 2022). Surface modification may improve the hydrophobicity of the cellulose; however, it might affect the hydrogen bonding capacities which results in low film quality.

Previously, many researchers used waste newspaper, old tissue paper, office paper and waste corrugated box to extract cellulose. The use of gloss art paper (GAP) waste which normally used for flyers and catalogue printing has rarely been reported. Therefore, in this study, the application of ultrasonic homogenization-assisted water pretreatment on the GAP waste is proposed to enhance the cellulose extraction, crystallinity, particle size quality, and its thermal stability. Optimization of pretreatment conditions (temperature, ultrasonic homogenization power level, and ultrasonic time treatment) was analyzed using Box–Behnken analysis. Surface modification of cellulose paper waste using TEMPO oxidation process and phosphoric acid hydrolysis was conducted. Cellulose–GAP waste film was generated after reinforced commercial starch for better hydrophobic with improved mechanical properties. The cellulose–GAP waste–starch (cGAP-st) film composite was tested for its mechanical properties, wettability analysis, thermal and functional group properties, transparency, and surface morphology. This study aimed to provide an understanding of the effect of the proposed treatment on the cellulose film from recycled GAP waste and contribute to research on biomass utilization for value-added products.

## Materials and methods

### GAP waste preparation

A gloss art paper (GAP) waste was cut into 2 cm × 2 cm in size to facilitate grinding process and soaked in distilled water with a ratio of 1:10 at different temperatures of 60, 75, and 90 °C for 2 h. After fully swelling, the GAP waste suspension was grounded into smaller particles using a Waring blender to provide uniform and small size of GAP waste and rinsed to remove the dirt or impurities yielding pulp GAP waste. The setup of the ultrasonic-homogenizer treatment started after the pulp GAP waste was mixed with distilled water at a 1:10 ratio and treated at different power intensity and varied time interval based on the experimental design. The treatment process was conducted in an air-conditioned room with samples in the open beaker to avoid in-situ heat access generated during the long homogenization process. After homogenization, the pulp GAP waste suspension was centrifuged and the remaining water was discarded.

### Experimental design using response surface methodology

The ultrasonic-homogenizer pretreatment of pulp GAP waste was done using the Ultrasonic-Homogenizer LUH 150 (Yamato Scientific Co., Ltd, Japan) at an oscillation frequency of 20 kHz ± 0.5 and energy input of AC100–240 V 1A single phase with a tapered microtip probe of 2 mm. The optimization was carried out using the Box–Behnken design in the statistical software package of RSM (Design-Expert 13.0.5.0, Stat Ease Inc. Minneapolis, USA). Three independent variables (temperature, power, and time) with three levels (−1,0, and 1) were selected based on preliminary experiment (refer Additional file 1: Table S1:Level of independent variables used in Box-Behnken design) were applied to the ultrasonic homogenizer to determine responses toward minimum particle size obtained, maximum crystallinity, and good thermal properties of the GAP waste after the treatment process. A total of 17 runs of randomly ordered experiments with 5 replications at the center point were performed and shown in Table 1.

**Table 1:** Effect of ultrasonic-homogenizer treatment on particle size, crystallinity, and thermal properties of GAP waste

Run	Variables			Responses		
	Temperature (°C)	Ultrasonic power (%)	Time (hour)	Particle size (nm)	Crystallinity (%)	Temperature decomposition (°C)
1	60	60	1.5	644.04	66.72	343.64
2	90	60	1.5	624.84	56.88	346.45
3	60	80	1.5	632.07	67.29	349.37
4	90	80	1.5	615.18	64.64	352.19
5	60	70	1.0	688.65	68.19	352.22
6	90	70	1.0	563.97	62.73	355.89
7	60	70	2.0	489.88	55.48	353.63
8	90	70	2.0	549.3	56.5	355.11
9	75	60	1.0	568.47	65.32	356.54
10	75	80	1.0	688.82	67.03	356.54
11	75	60	2.0	585.63	63.41	356.50
12	75	80	2.0	624.65	63.91	350.76
13	75	70	1.5	501.91	71.10	355.09
14	75	70	1.5	560.99	70.27	354.39
15	75	70	1.5	568.98	73.02	355.86
16	75	70	1.5	448.27	71.08	354.97
17	75	70	1.5	501.83	72.09	355.62

The regression analysis was used to optimize the independent variables and their interaction. For optimal point prediction, the second-order polynomial model was fitted to the response data as follows:

1 $$Y={\beta }_{0}+{\sum }_{i=1}^{3}{\beta }_{i}{X}_{i}+\sum_{i=1}^{3}{\beta }_{ii}{X}_{i}^{2}+ \sum_{i<j}^{3}{\beta }_{ij}{X}_{i}{X}_{j}$$

where Y is the predicted response, β_*0*_ is the model constant, β_*i*_, β_*ii*_ and β_*ij*_ are the linear, interaction, and quadratic coefficients, respectively; X_*i*_ and X_*j*_ are the independent variables in which *i* and *j* range from 1 to k (*k* = 3 in this experiment).

Analysis of variance (ANOVA) with a 95% confidence level was conducted for each response to test the models significant. A probability of *p* < 0.05 was used to evaluate the significant interaction between the independent variables, whereas the adequacy of the model was expressed by the coefficient of determination, R^2^.

### Film regeneration

Regenerated cGAP-st film was obtained and compared using two different cellulose surface modification methods (H_3_PO_4_ hydrolysis and TEMPO oxidation) to allow film formation with starch. In the surface modification of cGAP using H_3_PO_4_ hydrolysis (cGAP_H3PO4_), about 10 g of ultrasonic-homogenizer-treated GAP waste was added into 100 mL of 30% of H_3_PO_4_ solution and stirred for 1 h in an ice bath until GAP waste agglomeration was completely dissolved. The mixture was rinsed with distilled water and centrifuged for a few times to remove the remaining acid. Meanwhile, for the 2,2,6,6-tetramethylpiperidine-1-oxyl (TEMPO) oxidation process (cGAP_TEMPO_), about 10 g of ultrasonic-homogenizer-treated GAP waste was added into 300 mL distilled water containing 0.1 g of TEMPO and 1 g of NaBr. Then, a 30 mL of 10% NaClO_2_ solution was slowly added to the mixture to reach pH 10 and continuously stirred in the ice bath for 5 h. The mixture was then centrifuged and rinsed with distilled water a few times until neutral pH was achieved. During cGAP-st film preparation, both treated GAP waste (1 g) was added into a 50 mL 25 wt.% glycerol–water aqueous solutions containing 2% of potato starch at a ratio of 1:1 ratio of GAP waste cellulose to starch. A suspension was stirred and heated at 75 °C for 1 h until completely gelatinized before being pre-cooled to 40 °C. The homogenized solution was poured into a 10 cm of silicon plate and dried at 30 °C for 24–48 h in a fan oven until constant dry weight was obtained.

### Characterization

#### Lignocellulosic component

The lignocellulosic content was determined using Association of Official Agricultural Chemicals protocol (AOAC, 1997). Lignin, cellulose and hemicellulose were analyzed via neutral detergent fibers (NDFs), acid detergent fibers (ADFs), and acid detergent lignin (ADL). One gram of dried samples was put in the filtering crucible with porosity 0 and transferred to hot extractor unit (Fibertech System, USA) with the addition of 100 mL of NDF solution. The mixture was boiled for 1 h and then cooled and filtered. The residue was washed and transferred to cold extraction unit of the Fibertech System. All the residue was rinsed with the acetone and dried in the vacuum oven. The residue was then rinsed with hot distilled water before being dried in the oven at 105 °C until constant weight achieved. The procedure was repeated for ADF analysis using the acid detergent solution. Then, the ADL analysis was conducted using the residues from ADL samples with 25 mL of 72% of H2SO4 solution and stirred for three times at interval of 1 h. The mixture was filtered and rinsed with distilled water before being transferred to oven at 105 °C for 3 h. The samples were cooled in desiccator and the weight was recorded. The residue was then transferred to the furnace at 530 °C for 3 h and then cooled in desiccator before measure the final weight. The NDF determination was used to measure of total cellulose, hemicellulose and lignin in the biomass, while ADF method was used to determine of cellulose and lignin component and finally lignin content was measured through ADL method. The hemicellulose content was calculated as NDF–ADF, while the cellulose content was calculated as ADF–ADL. Lignin content was equivalent to ADL (Omar et al. 2017). All experiments were performed in triplicate.

#### Particle size

The particle size distribution was obtained using a dynamic light scattering analyzer (DelsaMax Pro 1.1.2.0 Beckman Coulter Life Sciences, USA). Approximately 0.01 g of GAP waste was suspended in 10 mL of distilled water and mixed using a vortex to avoid particles agglomeration. The suspension was added into the experimental cell and data acquisition was adjusted at 5 s of analysis time, laser wavelength of 532 nm, and scattering angle, *θ* = 163.5°. The apparent diameter of the GAP waste particle was characterized by the volume diameter distribution. Measurements were performed in triplicate.

#### X-ray diffraction (XRD)

The crystallinity was calculated from X-ray diffraction patterns using the SmartLab X-ray Diffractometer (Rigaku Co., Japan) at 40 kV and current 20 mA. Data were collected at 2*θ*, and the crystallinity index (CrI) was calculated using the Segal’s equation based on the diffraction pattern intensity. The peak intensity was in agreement with Segal’s peak height pattern in which *I*_200_ is the intensity of the higher peak in the diffractogram, while *I*_am_ is the minimum intensity which corresponded to the amorphous region of the diffractogram (Hafid et al. 2021).

2 $$\mathrm{CrI} \left(\%\right)=\frac{{I}_{200}-{I}_{\mathrm{am}}}{{I}_{200}} \times 100$$

#### Thermal properties analysis

Thermogravimetric (TG) and derivative thermogravimetric (DTG) analyses were conducted using a thermogravimetric analyzer (EXSTAR TG/DTA 6200, Seiko Instruments, Inc., Japan). The analysis was performed under a constant inert nitrogen flow at a rate of 100 mL/min. Samples weighing 5–9 mg were prepared and heated at 10 °C/min in a temperature range of 30–600 °C. Weight loss percentage (%) was determined from TGA curves, where the initial degradation temperature (*T*_onset_) and peak temperature (*T*_peak_) were defined as 1% weight loss from TGA curves, and the end set weight percentage was determined at 600 °C. The decomposition temperature was determined as the substances decomposed at higher degradation rate based on the DTG curves obtained.

#### Fourier transform infrared (FTIR) spectroscopy

Infrared spectra of the samples were analyzed to check the changes in the functional groups using Nicolet iZ10 (Thermo Fischer Scientific, Waltham, MA, USA) equipped with a golden gate diamond attenuated total reflectance (ATR) 10500 module with a germanium crystal using the single-reflection ATR method. Each spectrum was acquired by averaging 32 scans per sample in the wavelength range of 400–4000 cm^−1^ at 4 cm^−1^ resolutions and further analyzed using the OMNIC spectrum software (Thermo Fisher Scientific).

#### Scanning electron microscopy (SEM) and energy dispersive X-ray spectroscopy (EDS)

The surface morphology and structure of the dried film were analyzed using a Hitachi S-3400 N scanning electron microscope (Hitachi, Japan) at an accelerating voltage of 5–15 kV. The films were air-dried and coated with gold palladium in a Hitachi E-1010 sputter coater (Hitachi), and the images were taken at the same magnification of 500x (Hafid et al. 2021). Energy-dispersive X-ray spectrometry (EDX–SEM) was performed to micro-analyze the remaining heavy metal and printing elements present on the cGAP-st composite film.

#### Tensile strength analysis

Tensile properties of cGAP-st film were conducted on a tensile tester IMC‐18E0 model machine (Imoto Machinery Co. Ltd, Kyoto, Japan) at a crosshead speed of 5 mm/min. The tensile strength analysis was performed with a molded dumbbell (MK-11205–09, Saitama, Japan) with dimension of 65 × 12 × 0.1 mm with the 4 mm width narrow section and 20 mm gauge length. The measurement of each sample was carried out at 23 °C using 5 replicates. All the measurements were analyzed using Pressa2 testing software (Mustapha and Andou 2021).

#### Surface wettability and hydrophobicity analysis

The contact angle of cGAP-st films was measured using DropMaster DMS-401, Kyowa Interface Science Co. Ltd, Japan meter equipped with a video camera and FAMAS Measurement software. The analysis was carried out at room temperature with average of 5 times with water droplet volume of 0.9–1 µL for each measurement. Paper waste cellulose starch film was placed onto the glass slides to allow equilibration with ambient temperature and humidity before the analysis took place. The contact angle measurement was analyzed using the FAMAS software.

#### Transparency and UV shielding

The optical transmittance spectra and opacity of the cGAP-st film were determined using UV–Vis Spectrophotometer (Thermo Scientific GENESYS 20) at a wavelength ranging from 200 to 800 nm with blank quartz cell as a reference. The measurement was performed in replicates by collecting data on the transmittance.

## Results and discussion

### Characterization of GAP waste

A GAP waste used for this study was characterized by 73.92% of cellulose content with hemicellulose and lignin content of 14.08% and 12%, respectively. Cellulose extracted using ultrasonic homogenization treatment was remained to the great extent (~ 80% of yield) as compared to other chemical treatment by Hietala et al. (2018); Liu et al. (2020) which produced 53.4% and 95.17% of cellulose, respectively. Meanwhile, the lignin content also decreased from 12 to 4.67% along the treatment. Hydrolysis and depolymerization of lignin and the weak hemicellulose–cellulose hydrogen bonding had release the celluloses fraction in the lignocelluloses. This ultrasonic irradiation with high energy and temperature generates cavitation which sufficient to loosen the texture of paper waste by dislocated the lignin fiber, hence, exposed the cellulose layer (Abdullah et al. 2016; Soontornchaiboon et al. 2016). Oliva et al. (2020) stated the effect of acid chemical which hydrolyze the cellulose fraction and facilitate rapid dissolution that cause damage to the fiber length. On the contrary, mechanical shear and cavitation resulting from the ultrasonic homogenization process which led to partial dissolution of paper waste pulp also disrupt the particle size of the fibers but did not severely occur. Kumari and Singh (2020) stated the effect of harsh pretreatment on the amorphous fraction of the cellulose producing residual cellulose with more crystalline properties that later affected the integration of cellulose as a filler with other composite materials. This shows the ability of ultrasonic homogenization treatment on GAP waste for high yield and cellulose recovery.

### Optimization of ultrasonic-homogenizer process condition on particle size, crystallinity, and thermal properties of GAP waste

#### Regression coefficient and statistical analysis of the model

**Fig. 1:**
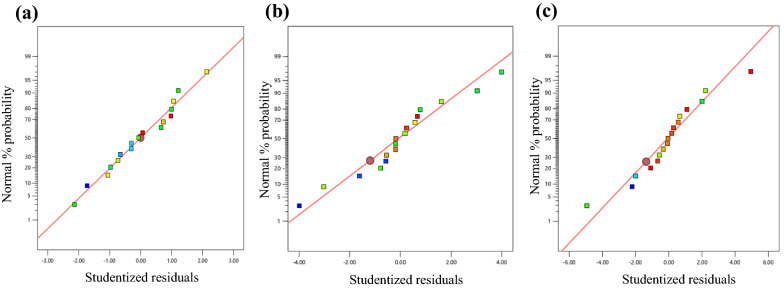
Normal probability of studentized result for **a** particle size with *R*^2^ of 0.76; **b** crystallinity with *R*^2^ of 0.91; and **c** thermal decomposition with *R*^2^ of 0.81 of the treated ultrasonic-homogenizer GAP waste treatment

The experimental results by the Box Behnken design were assessed by ANOVA to determine the treatment of ultrasonic-homogenizer effects toward the particle size, crystallinity, and good thermal properties of GAP waste. Regression coefficients of the predicted second-order polynomial models are summarized in Table 2. The statistically significant independent variables were considered in the probability *p* < 0.1, which the regressions were considered significant at 90%. The *F* value of particle size, crystallinity, and temperature decomposition were 2.57, 8.04, and 3.24, respectively, which are significant, as there was only 11.34%, 0.59%, and 6.79% (of each response) chance of model *F* value could occur due to noise. The lack of fit (LoF) for all the dependent variables was insignificant (*p* > 0.05). The goodness of fit of the polynomial model was examined based on the coefficient of determination (R^2^) between 0.76 and 0.91, which is in good agreement with the experimental and predicted values. The lower coefficient of variation (CV) of 0.71–8.62 values suggested the relationship of ultrasonic-homogenizer treatment of GAP waste and responses were well-described by the model. The fitted model was further verified for its adequacy by plotting the normal probability plot distribution graph of particle size, crystallinity, and temperature decomposition, as shown in Fig. 1. The residual positive probability distribution explained the imitative effects of the model surface as the closer the expansion rate toward the probability distribution, the higher reliability of the model in predicting the optimum parameter and responses during the treatment process (Yang et al. 2020). The statistical analysis results can be used for the prediction of the optimized parameters of the GAP waste cellulose production process.

**Table 2:** ANOVA table of ultrasonic-homogenizer-treated paper waste on process responses (particle size, crystallinity and thermal properties) *F* value

Source	Particle size (nm)					Crystallinity (%)					Temperature decomposition (°C)				
	Sum of square	DF	Mean square	*F* value	*p* value	Sum of square	DF	Mean square	*F* value	*p* value	Sum of square	DF	Mean square	*F* value	*p* value
Model	57,749.72	9	6416.64	2.57	0.1	428.79	9	47.64	8.04	0.0059	181.29	9	20.14	3.24	0.0679
A: Temperature (°C)	1283.98	1	1283.98	0.51	0.4966	35.83	1	35.83	6.05	0.0435	14.53	1	14.53	2.33	0.1705
B: Power (%)	2371.54	1	2371.54	0.95	0.3623	13.89	1	13.89	2.34	0.1696	4.1	1	4.1	0.66	0.4436
C: Time (hour)	8479.28	1	8479.28	3.4	0.1079	71.82	1	71.82	12.12	0.0102	3.37	1	3.37	0.54	0.486
A^2^	4967.23	1	4967.23	1.99	0.2013	147.12	1	147.12	24.83	0.0016	69.85	1	69.85	11.22	0.0123
B^2^	25,807.35	1	25,807.4	10.33	0.0148	12.43	1	12.43	2.1	0.1907	43.13	1	43.13	6.93	0.0338
C^2^	2076.42	1	2076.42	0.83	0.3922	100.11	1	100.11	16.9	0.0045	40.45	1	40.45	6.5	0.0382
AB	1.33	1	1.33	0.0005	0.9822	12.92	1	12.92	2.18	0.1832	2.50E-05	1	2.50E-05	4E-06	0.9985
AC	8473.2	1	8473.2	3.39	0.108	10.5	1	10.5	1.77	0.2249	1.2	1	1.2	0.19	0.674
BC	1653.64	1	1653.64	0.66	0.4426	0.37	1	0.37	0.062	0.8108	8.24	1	8.24	1.32	0.2878
CV (%)	8.62					3.71					0.71				
*R*^2^	0.76					0.91					0.8062				

*p* < 0.1 significant; *p* < 0.05 highly significant

#### Response surface analysis

**Fig. 2:**
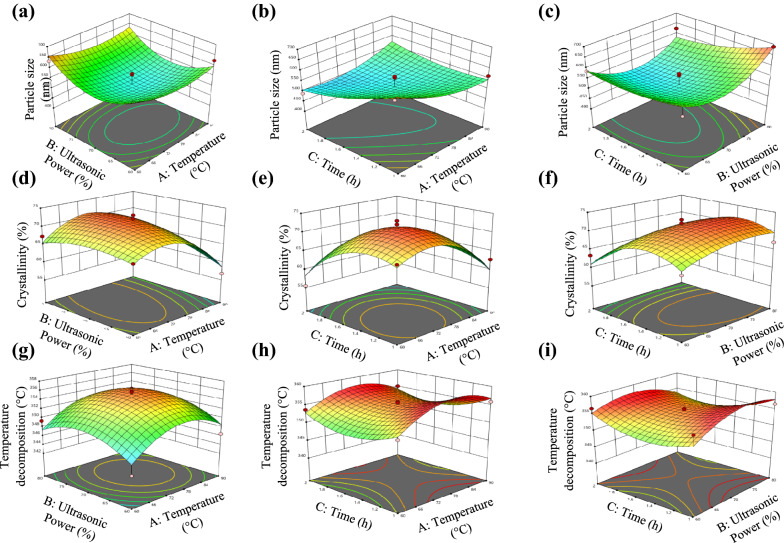
Response surface plots depicting the interaction of **a** temperature and ultrasonic power, **b** temperature and time, **c** ultrasonic power and time on particle size of cellulose, **d** temperature and ultrasonic power, **e** temperature and time, **f** ultrasonic power and time on crystallinity of cellulose, **g** temperature and ultrasonic power, **h** temperature and time, and **i** ultrasonic power and time on temperature decomposition of paper waste cellulose

The interaction of independent variables on particle size, crystallinity, and temperature decomposition of pulp GAP waste in a 3D response surface plot is illustrated in Fig. 2. The influence of temperature, ultrasonic-homogenizer power or intensity, and treatment time of particle size (Fig. 2a–c) can be reflected from the 3D surface of the graph with the minimum value of the response showed the lowest particle size of the pulp GAP waste achieved after the treatment process. The particle size reduced significantly from 688 to 448 nm with the increased in temperature (75 °C), ultrasonic-homogenizer power level of 70%, and treatment time (1.5 h) implying that the effect of particle size depended on all the variables studied. Defibrillation of pulp GAP waste with the aid of ultrasonication process breaks the microfibrils into individual fibers produced uniformly dispersed nanosized fibers (Asem et al. 2021). Cellulose fiber dissolution was achieved through the energy generated by ultrasonication in which the hydrogen bond gradually disintegrated the aggregations of the cellulose fibers (Mohd Ishak et al. 2020).

Meanwhile, both temperature (*p* < 0.04) and treatment time (*p* < 0.01) are the most governing factors for the increment of crystallinity index (CrI) of the treated pulp GAP waste (*p* < 0.01) followed by ultrasonic power level factors (Fig. 2d–f). The CrI of treated pulp GAP waste is one of the important features influencing the mechanical and thermal properties. Increasing temperature and extending the ultrasonication time intensified the treatment and enhanced the disruption of the amorphous region, thus exposed the crystalline fraction of the treated pulp GAP waste. Increment in crystallinity from 57.23% to 71.85% was observed at 75 °C with 1.5 h time using 70% of power level. The decreasing trend of CrI was observed at higher temperatures with extended ultrasonication treatment time. The CrI was reduced from 70.3% to 56.8% with the increase of temperature from 75 to 90 °C and sonication time up to 2 h (Fig. 2f). Temperature provides heat energy, which enhances the protonation reactions (Bello and Chimphango 2021) and facilitates the action of ultrasonic homogenizer in breakage of amorphous region for high crystallinity of pulp GAP waste. At longer treatment time, ultrasonication with high energy break the intermolecular hydrogen bonds of pulp GAP waste cellulose and become non-selective by removing both amorphous and crystalline regions causing the collapse of crystal structure that explained the reduction in CrI of treated pulp GAP waste.

The increase in treated pulp GAP waste thermal stability from 345.8 to 355.3 °C with increase in temperature to 75 °C and power level of 70% was expected from ultrasonic pretreatment (Fig. 2g–i). The increase in thermal stability of the cellulose was directly proportional to the CrI values. As the high amorphous region was sensitive toward high-temperature degradation, the crystalline fraction was on contrary contributed to the greater thermal properties of the cellulose (Yacob et al. 2019; Qu et al. 2021). It was observed that the extent of thermal stability gradually increased with the treatment temperature from 60 to 75 °C and ultrasonic power of 60–70%. However, as the incubation time increased, the treated pulp GAP waste showed a reduction in its thermal properties, in which attributed to the depolymerization and decomposition of the glycosidic linkages of cellulose and the formation of char residue (Kassem et al. 2020). Within this range, the cellulose crystal was loosened, and rearrangement of cellulose crystallite occurred yielding a more compact crystal structure with higher decomposition temperature. Nevertheless, if the ultrasonic time was taken into consideration, a de-structuring process of cellulose of treated pulp GAP waste might take place at a longer treatment time thus will lower the crystallinity and lead to easier degradation of cellulose and observation of low degradation temperature (Mohd Ishak et al. 2020).

#### Validation of pretreatment condition

While keeping the independent variables (temperature, ultrasonic power, and treatment time) within the set range, the optimum ultrasonic homogenization treatment conditions were determined by minimizing the response of particle size and maximizing the crystallinity index and temperature decomposition of the pulp GAP waste (Table 3). The optimum value of the particle size, CrI, and temperature decomposition predicted by the numerical RSM model was 516.4 nm, 71.51%, and 355.20 °C, respectively, at 75.82 °C with 70.27% of ultrasonic power level for 1.42 h of treatment conditions with a high desirable function of 0.84.

**Table 3:** Optimization parameter limit (actual and optimized) values and desirability toward responses (particle size, crystallinity, and thermal properties)

Variables	Target	Experimental limit	Optimum value	Desirability
Model				0.84
A: Temperature (°C)	In range	60–90	75.80	
B: Power (%)	In range	60–80	70.27	
C: Time (hour)	In range	1–2'	1.42	

Response		Experimental range	Predicted	Actual
Particle size (nm)	Minimum	448.27–688.82	516.4	522.03
Crystallinity (%)	Maximum	55.48–73.02	71.51	71.76
Temperature decomposition (°C)	Maximum	343.64–356.54	355.19	355.47

A validation experiment was conducted using the predicted treatment conditions and the obtained results of 522.03 nm of particle size, 71.76% of CrI, and decomposition temperature of 355.50 °C agreed with the value of RSM regression study. This suggested the reliability of the Box–Behnken experimental design in optimizing and understanding the individual and interaction effect of the parameters during the ultrasonic homogenization treatment process of paper waste.

### Composite cellulose GAP waste reinforced starch (cGAP-st) film

Ultrasonic-homogenizer paper waste comprises 70–80% of cellulose content with smaller particle size with acceptable values of crystalline and thermal resistance properties. However, owing to the reduction in fibers quality and hydrophilicity properties, its potential to be recycled is inevitably difficult. The adsorption of water from the environment due to hydrophilicity features decreases the efficiency of the cellulose barrier’s properties against water and gasses, especially for its application as food packaging film materials (Balasubramaniam et al. 2020). Therefore, the cellulose–GAP waste was modified using H_3_PO_4_ (cGAP_H3PO4_) hydrolysis and TEMPO (cGAP_TEMPO_) oxidation before being reinforced with starch (hereafter known as cGAP_H3PO4_-st and cGAP_TEMPO_-st) to improve the film hydrophobicity, strength, and quality. The dissolution mechanisms of paper waste cellulose in H_3_PO_4_ were initiated by the esterification reaction between H_3_PO_4_ and the hydroxyl group of cellulose in which the hydrolysis reaction occurred yielding the regenerated cellulose (Kassem et al. 2020). Incorporating and impregnation of cellulose with starch is a suitable approach owing to its similar structure and could attain poor mechanical, physical, and chemical properties of starch as a composite film for the packaging industry (Liu et al. 2021).

#### Surface characteristic and wettability analysis

**Fig. 3:**
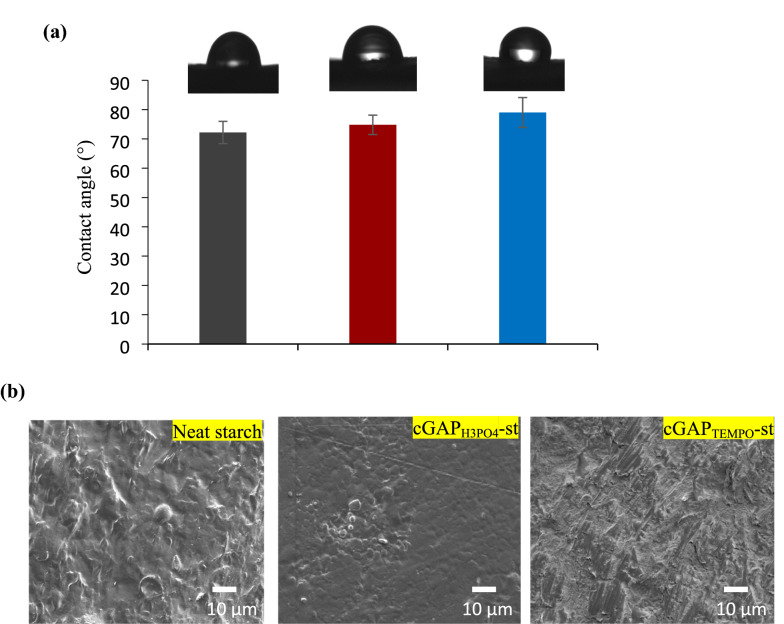
Photograph of **a** Contact angle and **b** SEM micrograph of the neat starch, cGAP_H3PO4_-st and cGAP_TEMPO_-st film

The wettability properties of neat starch, cGAP_H3PO4_-st and cGAP_TEMPO_-st films were determined by contact angle analysis (Fig. 3). Wettability is the ability to withhold liquid on the surface of the composite films, which also determines the hydrophilicity properties of the films (Megashah et al. 2020). Cellulose-based coating films have the ability to absorb moisture and swell at relatively high humidity that influence the barrier performance as packaging materials (Nuruddin et al. 2021). The contact angle of the neat starch film was 72.2° and the contact angle of cGAP_H3PO4_-st and cGAP_TEMPO_-st films increased to 74.8° and 79°, respectively, corresponded to an improvement of 4–10% (Fig. 3a).

Increased in the degree of contact angle indicates a reduction in hydrophilicity of both cGAP_H3PO4_-st and cGAP_TEMPO_-st films attributed to the action of phosphoryl and carboxyl groups during dissociation of H_3_PO_4_ hydrolysis and TEMPO oxidation process, which occupy a large number of hydroxyl group of the cellulose and starch structure. The hydrophobic backbones were exposed, and the hydrophilic hydrocarbon region of the film matrix was removed which was reflected by the increase in contact angle degree (Chowdhury 2019). The TEMPO-mediated oxidation process was targeted to separate the paper waste fibers and facilitate its mechanical disintegration (Kassab et al. 2020). The strong interfibrils interactions was favored by the oxidized groups with a few •OH available (Chiulan et al. 2021). In addition, cellulose GAP waste might contain a trace amount of lignin that contributed to an increase in contact angle of the starch–cGAP network, as lignin is more hydrophobic than cellulose (Hietala et al. 2018). However, it did not exhibit a significant difference as compared to the number of hydroxyl group in the structure network.

The entanglement of neat starch, cGAP_H3PO4_-st and cGAP_TEMPO_-st were observed using SEM (Fig. 3b). A strongly bonded fiber with densely packed composite film was observed due to the close intertwining of the cellulose GAP fiber with the starch material (Megashah et al. 2020). In comparison with neat starch film, cGAP_H3PO4_-st and cGAP_TEMPO_-st film exhibited a flat and slightly smoother surface with fewer voids. The cGAP_TEMPO_-st film revealed micro and nano-size porous structures and bumpier surface as compared to cGAP_H3PO4_-st that are rather even and spotless. These occurrences may be due to mild process condition of cellulose treatment using H_3_PO_4_ as compared to TEMPO. Cellulose GAP waste with smaller diameter and chain length was generated after the H_3_PO_4_ hydrolysis and TEMPO oxidation process. This treatment selectively converts the C6 hydroxyl group of cellulose to carboxylate group, providing H_3_PO_4_/TEMPO-oxidized cellulose with high reactivity and facilitating the graft of low molecular weight compounds (Chiulan et al. 2021). In addition, Patino-Maso et al. (2019) reported the TEMPO oxidation process separates the fiber bundles of the cellulose into thinner fibers which lead to the decrease of the molecular weight of the cellulose chain and yield the shorter chain of the fiber length. The alteration on surface morphologies demonstrated that cGAP_H3PO4_-st and cGAP_TEMPO_-st were homogeneously incorporated within the starch matrix and provided more compact structure as compared to neat starch. Since there was no clear discern individual cellulose GAP waste, the length and diameter of the fiber structures were not measured during the SEM studies.

#### Mechanical properties

**Fig. 4:**
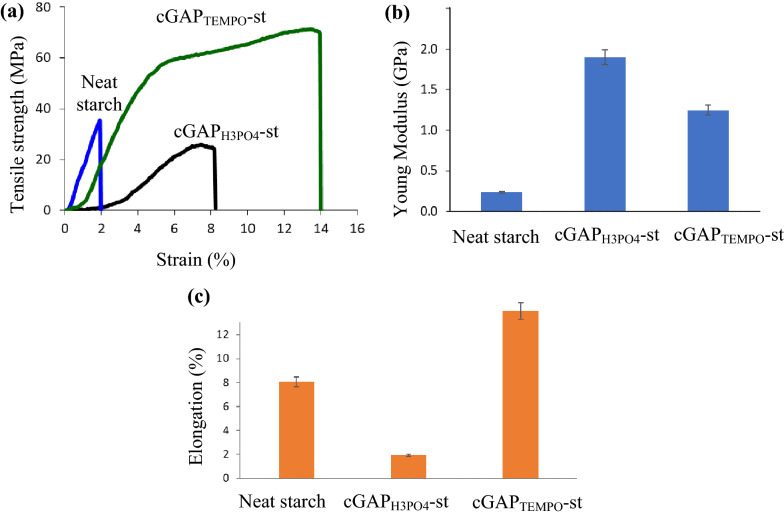
Mechanical properties of starch–cellulose paper waste: **a** representative stress–strain curves; **b** Young Modulus; and **c** elongation at break for starch cellulose paper waste treated with H_3_PO_4_ and TEMPO

Figure 4 demonstrates the mechanical properties of neat starch, cGAP_H3PO4_-st and cGAP_TEMPO_-st. The stress–strain curves exhibited an extraordinary combination of high tensile (71.32 MPa) and elongation (up to 13%) for cGAP_TEMPO_-st film sheet which was higher than the corresponding value of neat starch and cGAP_H3PO4_-st_._

Higher tensile strength was attributed to the surface modification of the hydroxyl group that increased the compatibility of the treated cGAP with the starch matrix (Takagi et al. 2013). The elongation-at-break and Young Modulus values were plotted in Fig. 4b and c. The cGAP_TEMPO_ shows high elongation without losing the tensile strength associated with the highly disordered of the fibers that formed interfibril and intrafibril hydrogen bonds linkages that allowed efficient sliding during tensile analysis. However, a slight reduced value of Young Modulus obtained was caused by the slippage and porosity of the composite as pores will act as energy dissipators under applied tension (Chen et al. 2020). The cGAP_H3PO4_-st composite showed tensile strength of 35.48 MPa with the highest Young Modulus of 1.9 GPa and elongation-at-break of 1.93%.

The decrease in tensile strength and elongation at break were due to the non-homogenous dispersion of cGAP_H3PO4_ in the starch matrix and the probability of agglomeration (Kassab et al. 2020). Despite reduction in tensile strength and elongation at break, the increment in Young Modulus value by more than 100% exhibited the higher stiffness as compared to the neat starch film. Stiffness of the cellulose matrix was contributed by the interfibril hydrogen bond density (Wang et al. 2015). It can be seen from the dense and packed fiber of cGAP_H3PO4_ from the SEM images correlated with the high interfibril hydrogen linkages, explaining the higher Young Modulus value as compared to the more porous structure of neat starch and cGAP_TEMPO_. Application of cellulose as fillers demonstrated high reinforcing ability with other matrix due to the nanometric size of the cellulose particles, the functionalized surface modification by solvent, and the aspect ratio of the cellulose fiber, which enhanced the support and interfacial interaction between the polymer chains that significantly increased its mechanical properties (Kassab et al. 2020).

#### XRD, FTIR spectroscopy and thermal stability analysis

**Fig. 5:**
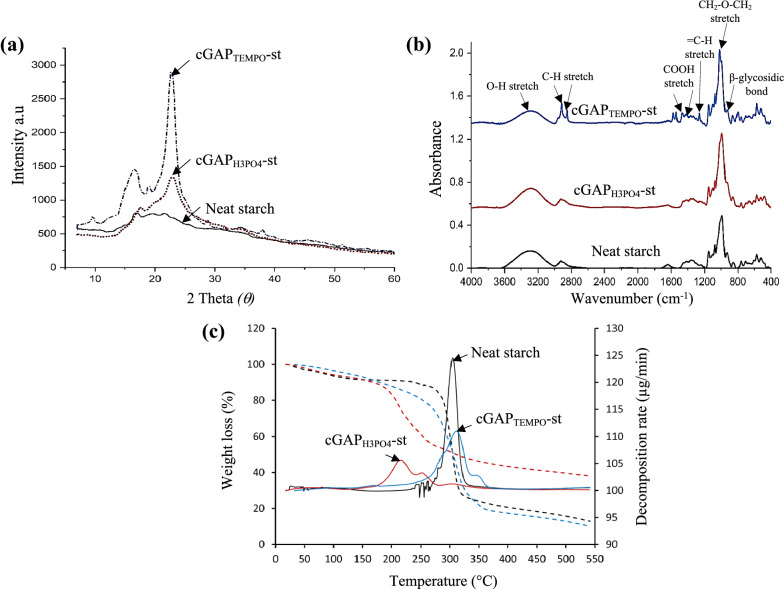
Profile of **a** XRD; **b** FTIR; and **c** thermal properties analysis of neat starch, cGAP_H3PO4_-st and cGAP_TEMPO_-st film

The neat starch, cGAP_H3PO4_-st and cGAP_TEMPO_-st films were evaluated for its crystallinity pattern using XRD analysis. As shown in Fig. 5a, the diffractograms at sharp peaks of 2*θ* = 17.8, 22.7 showed a typical crystal lattice of cellulose II polymorph with CrI of St-Cel_PW_-H_3_PO_4_ and St-Cel_PW_-TEMPO were calculated as 59.6% and 47.66%, respectively. A reduction in CrI by 16–33.5% was observed after surface modification processes by H_3_PO_4_ and TEMPO owing to the removal of amorphous and partly of crystalline components due to the strong chemical reactions. Nigam et al. (2021) reported on high CrI was observed due to the removal of amorphous, which was regarded as hemicellulose and lignin structures during the treatment process. Compared with the original starch film, XRD pattern of the cGAP_H3PO4_-st and cGAP_TEMPO_-st revealed a localization change of the main diffraction peaks at 22.2°, corresponding to the crystallographic planes of cellulose II, which was a more stable structure for mechanical strength. This was in agreement with Kassem et al. (2020) who observed a great impact of the solvent selection on CrI values. The sharp decreased may be due to the breakage of hydrogen bonding among the hydroxyl group of the cellulose and substituted by negatively charge phosphoric groups and carboxyl group moiety on the surface of the cellulose backbone (Joshi et al. 2019). Plus, acid hydrolysis and TEMPO oxidation process disrupted the intermolecular chain interactions, thus caused swelling of the crystalline region of cellulose in treated paper waste (Ramadoss and Muthukumar 2015). Crystallinity signified an active and stabilized surface charge suspension of cGAP_H3PO4_-st and cGAP_TEMPO_-st, which led to a more homogenized mixture and facilitated uniform network contributed to the good mechanical properties of the starch–cGAP film.

In the FTIR analysis (Fig. 5b), the prominent peak at 3292 cm^−1^ was attributed to the stretching vibration of O–H hydrogen bond, normally associated with the strength of the particles, whereas the two associated peaks at 2915 cm^−1^ and 2846 cm^−1^ indicated CH_2_ stretching vibration which is the distinguished features of cellulose (Ramadoss and Muthukumar 2015; Rosli et al. 2021). The peak absorption at 1649 cm^−1^ belonged to O–H stretching vibration of water, which characterized the hydrophilic features of the cellulose samples. Two intensified peaks (1538 cm^−1^ and 1543 cm^−1^) were attributed to the asymmetric stretching vibration of COOH groups that was only shown in cGAP_TEMPO_-st. A similar observation was reported by Qu et al. (2021) on the overlapping peaks during TEMPO oxidation and the O–H bending vibration of the absorbed water. The bands at 1259 cm^−1^, 1364 cm^−1^, 1366 cm^−1^, and 1433 cm^−1^ in the spectrum were related to the symmetric bending of CH_2_ in which the specific band between 1415 and 1420 cm^−1^ was attributed to the crystalline structure of the cellulose (Joshi et al. 2019). The FTIR spectrum of two peaks at a shoulder of 1085 cm^−1^ showed − CH_2_ − O − CH_2_ − stretching of the methyl group characteristics and particularly prominent in cGAP_TEMPO_-st, implying methyl group presence from TEMPO oxidation process, whereas peak at 1056 cm^−1^ was assigned to a skeletal vibration of the C–O–C pyranose ring present in cellulose (Baruah et al. 2020). Cellulose characteristic band at a sharp peak at 992 cm^−1^ depicts the cellulose spectrum corresponding to β-glycosidic bond, between the sugar units in hemicellulose and cellulose (Ramadoss and Muthukumar 2015). Meanwhile, the peak at 900 cm^−1^ was assigned to the amorphous region of cellulose, was similarly reported by Poletto et al. (2014). No other derivational functional group appeared during the H_3_PO_4_ hydrolysis and TEMPO oxidation process of cellulose paper waste.

The TGA and DTG curves of the neat starch, cGAP_H3PO4_-st and cGAP_TEMPO_-st which determined the thermal properties of starch–cellulose films are presented in Fig. 5c. In general, two phases of thermal degradation patterns were observed between 30 and 150 °C that corresponded to the initial weight loss due to the water and other volatile organic matters evaporation that were contained in the starch–cellulose matrix. Then, it was followed by polymer degradation temperature at 250–400 °C, which attributed to the cellulose, hemicellulose, and lignin fraction (Kassab et al. 2020). The cGAP_TEMPO_-st showed a higher decomposition temperature (T_max_) of 313.5 °C and a maximum weight loss (63.31%) as compared to the neat starch film (306.3 °C) and cGAP_H3PO4_-st (217.4 °C). Meanwhile_,_ the rate of film decomposition showed in DTG peaks exhibited rapid decomposition of neat starch film (124.1 µg/min) as compared to cGAP_H3PO4_-st (105.6 °C) and cGAP_TEMPO_-st (110.9 µg/min) at a higher temperature. The T_max_ of cGAP_TEMPO_-st was 11.7%, slightly lower than cellulose–paper waste after ultrasonic homogenization process and it may be due to the crystallinity reduction, suggesting a greater loss in thermal stability of the cellulose. In addition, hydrogen bond intermolecular chains breakage during TEMPO oxidation process and the OH group substitution of the cellulose with the carboxylic groups from the TEMPO catalyst are the possible causes of the decrease in decomposition temperature of the cGAP_TEMPO_-st. Meanwhile, cGAP_H3PO4_-st had a lower decomposition temperature with 48.2% of weight loss as compared to the starch film control sample due to the functionalized surface. Dissolution of cGAP in the H_3_PO_4_ induced a rearrangement and reformation of its hydrogen bonding network with respect to grafted phosphate groups (Kassem et al. 2020). In addition, formation of higher char was observed and was due to the presence of phosphate groups characterized by its flame retardant properties due to its ability in char formation (El Achaby et al. 2018).

#### Transparency and UV shielding

**Fig. 6:**
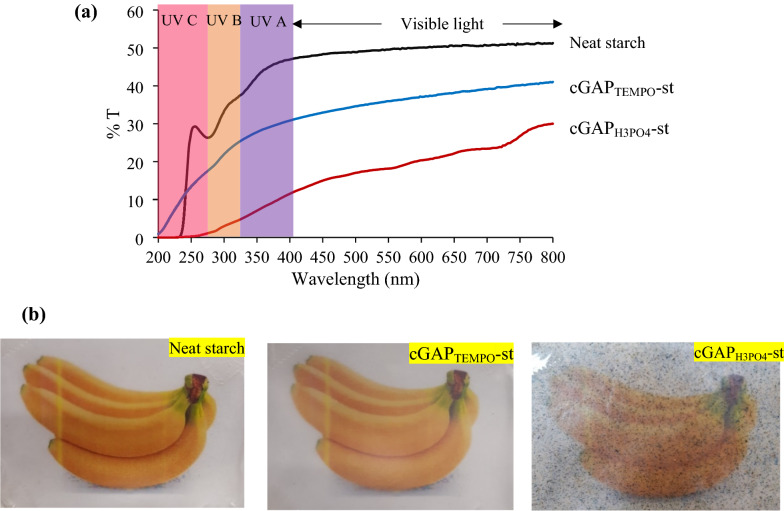
Optical properties of starch–cGAP composite film: **a** UV–Vis light transmittance spectra, and **b** visual appearance of (i) neat starch film; (ii) cGAP_TEMPO_-st and; (iii) cGAP_H3PO4_-st

Transparency of coated packaging materials originated from the nanofiber size homogeneity and degree of fibrillation between the cellulose paper waste with starch matrix components. Transparency of the film materials depends on the diffuse transmittance of wide and narrow-angle light scattering which plays a role in customer’s visual inspection of the products (Nuruddin et al. 2021). In Fig. 6a, the neat starch, cGAP_H3PO4_-st, and cGAP_TEMPO_-st films transmittance spectra were determined and the average percentage of transmittance in the UV–Vis region (UV A: 320–400 nm; UV B: 280–320 nm; and UV C: 190–280 nm). The neat starch film displayed a high transmittance in both visible light area (transmittance of 51.1%) and UV–Vis region, UV A, UV B, and UV C of 46.9%, 36.8%, and 29.3%, respectively. The cGAP_TEMPO_-st films with moderate transparency showed a decrease in transmittance (40.9%) in visible light area and transmittance reduction of 32–38% in the UV–Vis region. The TEMPO treated cGAP waste produced nano particle size, negatively charge groups, good dispersion, and better interfacial interaction with starch macromolecular chains, yielding better quality of transparent film as compared to cGAP_H3PO4_-st. The lowest transparency was observed in cGAP_H3PO4_-st with only 30% of transmittance detected in the visible light area, whereas the transmittance results of UV A, UV B, and UV C were 11.5%, 4.4%, and 1.3%, respectively, due to agglomeration and poor dispersion of cGAP_H3PO4_ in the starch matrix (Kassab et al. 2020). All synthesized films displayed smoother surfaces and high clarity in the order of neat starch > cGAP_TEMPO_-st > cGAP_H3PO4_-st (Fig. 6b).

The TEMPO-oxidized treatment cellulose paper waste was attributed to smaller size with high homogeneity and led to a higher transparency of cGAP_TEMPO_-st films. Kaffashsaie et al. (2021) explored on the electro-statistical forces created by TEMPO oxidation process to produce elementary fibrils of individual and bundles form, thus produced better dispersion and avoided agglomeration in the cGAP_TEMPO_-st films. Meanwhile, the cGAP_H3PO4_-st film with poor transparency was attributed by the presence of residual ink of the paper waste that was incompletely removed by only using the H_3_PO_4_ hydrolysis treatment. A high transparency of the cGAP_TEMPO_-st film showed its positive effect and suitable candidate as a packaging application material. The above results indicated that the proposed process for cellulose extraction for packaging purposes is technically feasible. However, more studies on the economic perspective and environmental impact need to be carried out for better understanding and improvement, especially during manufacturing process.

## Conclusion

Cellulose has been successfully extracted from gloss art paper waste using ultrasonic homogenization process. The particle size of cellulose pulp GAP waste was reduced, and the crystallinity and thermal resistance temperature were increased at optimized temperature of 75.8 °C, 70.27% power level, and 1.42 h of time, resulting in particle size of 516.4 nm, 71.51% crystallinity index, and 355.2 °C of thermal resistance. The ultrasonic-cellulose GAP extract was treated using surface modified method with phosphoric acid and TEMPO oxidation process before being reinforced with starch polymers for composite film generation. Both surface modification approaches resulted in an increase in surface hydrophobicity that affected the wettability properties of the starch–cellulose composite film. The increase in tensile strength of the cGAP_TEMPO_-st and cGAP_H3PO4_-st composite films to 71.32 MPa and 35.48 MPa was obviously high as compared to neat starch (25.83 MPa), indicating that the homogeneous dispersion of surface treated cellulose increased the compatibility with the starch matrix. The highest crystallinity of the films was recorded by cGAP_H3PO4_-st, followed by cGAP_TEMPO_-st, and neat starch. An improvement in temperature degradation was observed with cGAP_TEMPO_-st and no observed reduction of temperature degradation in cGAP_H3PO4_-st film due to the functionalized surface of the chemicals. Transparency analysis results exhibited a higher light transmittance for the starch–cellulose GAP TEMPO film as compared to starch–cellulose GAP–H_3_PO_4_, but was slightly lower than the neat starch. Surface modification of the cellulose paper waste treated ultrasonic definitely improved the starch–cellulose composite films for further application as packaging material.

### Supplementary Information

**Additional file 1: Table S1. **Level of independent variables used in Box–Behnken design.

## Data Availability

The data used in this study are available upon reasonable request from the corresponding author.
