# Supplementary material for: Extraction and surface modification of cellulose fibers and its reinforcement in starch-based film for packaging composites
Source: Bioresour Bioprocess. 2023 Jan 25;10(1):7. doi: 10.1186/s40643-023-00631-w (PMC10992750; doi:10.1186/s40643-023-00631-w)
Supplement: Supplementary file 1 — Additional file 1: Table S1. Level of independent variables used in Box–Behnken design. [file 40643_2023_631_MOESM1_ESM.docx]

**Extraction and surface modification of cellulose fibers and its reinforcement in starch-based film for packaging composites**

Halimatun Saadiah Hafid^a,*^, Farah Nadia Omar^b,*^, Ezyana Kamal Bahrin^c,d^,Minato Wakisaka^e^

^a^Institute of Plantation Studies, Universiti Putra Malaysia, 43400 UPM Serdang, Selangor, Malaysia

^b^Preparatory Center for Science and Technology (PPST), Universiti Malaysia Sabah, Jalan UMS, 88400 Kota Kinabalu, Sabah, Malaysia

^c^Institute of Plantation Studies, Universiti Putra Malaysia, 43400, UPM Serdang, Selangor, Malaysia

^d^Centre of Foundation Studies for Agricultural Science, Universiti Putra Malaysia, 43400, UPM Serdang, Selangor, Malaysia

^e^Graduate School of Life Science and Systems Engineering, Kyushu Institute of Technology, 2-4 Hibikino, Wakamatsu-ku, Kitakyushu, 808-0196, Japan

Corresponding authors:

[farahnadiaomar@ums.edu.my](mailto:farahnadiaomar@ums.edu.my)

[halimatun@upm.edu.my](mailto:wakisaka@life.kyutech.ac.jp)

**SUPPORTING INFORMATION**

**List of Tables**

Table S1 Level of independent variables used in Box-Behnken design

**Table S1.** Level of independent variables used in Box-Behnken design

| Variables | Coded level | | |
| --- | --- | --- | --- |
|  | Low (-1) | Center (0) | High (1) |
| Temperature (°C) | 60 | 75 | 90 |
| Ultrasonic power (%) | 60 | 70 | 80 |
| Time (hour) | 1.0 | 1.5 | 2.0 |
